# Learner satisfaction with synchronous and asynchronous virtual learning systems during the COVID-19 pandemic in Tehran university of medical sciences: a comparative analysis

**DOI:** 10.1186/s12909-023-04872-3

**Published:** 2023-11-21

**Authors:** Hossein Dargahi, Mahdi Kooshkebaghi, Masoumeh Mireshghollah

**Affiliations:** 1 Health Management, Policy Making and Economic Department, School of Public Health, Health Information Management Research Center, Tehran University of Medical Sciences, Tehran, Iran; 2 Health Services Management, Yas Hospital, Tehran University of Medical Sciences, Tehran, Iran; 3 Educational Management, School of Nursing and Midwifery, Tehran University of Medical Sciences, Tehran, Iran

**Keywords:** Electronic learning, Learners, COVID-19 pandemic, Online and offline systems, Tehran University of Medical sciences

## Abstract

**Background:**

The need for electronic learning and its systems, especially during specific circumstances and crises, is crucial and fundamental for users in universities. However, what is even more important is the awareness and familiarity of learners with different systems and their appropriate use in e-learning. Therefore, the present study was conducted to determine the satisfaction of learners with synchronous and asynchronous electronic learning systems during the COVID-19 period at Tehran University of Medical Sciences.

**Methods:**

The present study was a descriptive-analytical study conducted cross-sectionally from the first semester of 2019–2020 academic year until the end of the second semester of 2021–2022 academic year, coinciding with the COVID-19 pandemic. The sample size was determined to be 370 students and 650 staff members using the Krejcie and Morgan table. The face validity and reliability of the research tool, which was a researcher-made questionnaire, was confirmed. Considering a response rate of 75%, 280 completed questionnaires were received from students, and 500 completed questionnaires were collected from employees. For data analysis, absolute and relative frequencies, as well as independent t-test, analysis of variance (ANOVA), and Post Hoc tests in the SPSS software were utilized.

**Results:**

During the COVID-19 pandemic, both students and staff members at Tehran University of Medical Sciences showed a relatively decreasing level of satisfaction with electronic learning. There was a significant difference in satisfaction between these two groups of learners regarding electronic learning (P = 0/031). Learners were relatively more satisfied with the offline system called “Navid” compared to online learning systems. Among the online systems, the highest level of satisfaction was observed with the Skype platform.

**Conclusion:**

Although learners expressed relative satisfaction with electronic learning during the COVID-19 period, it is necessary to strengthen infrastructure and provide support services, technical assistance, and continuous updates for electronic learning platforms. This can contribute to more effective and efficient utilization of electronic learning, especially during particular circumstances and crises, or in hybrid models combining online and face to face education and training.

## Background

Over recent decades, electronic learning (or e-learning) has been put into effect to overcome the main learning challenges faced in the 21st century, and weigh up the Internet development as well as the exploitation of Internet-based technologies across the world. At present, such dynamic and intelligent changes can be detected at universities and within their research interests, alongside much emphasis by researchers and learner satisfaction, and their impact on e-learning systems based on some criteria [1]. However, what matters is user’s awareness of different types of e-learning systems and making the correct choice and proper utilization of them. Therefore, e-learning is not about bringing people to learning, but bringing learning to people [2].

In the present day, the field of teaching and learning has undergone significant transformations facilitated by information technology. The primary application of information technology in this regard is e-learning, which manifests in diverse forms such as computer-based learning, network-based learning, and web-based training [3].

Learner satisfaction with online education has been documented as one of the key components in e-learning. Alqurashi, recruiting 167 students, had accordingly reported that e-learning self-efficacy, learner-content connection (LCT), and learner-learner interaction (LLI) could be among the factors contributing to high levels of satisfaction with e-learning among these individuals based on the regression analysis outcomes. Therefore, educators had been suggested to consider online learning self-efficacy (OSLE), LCT, and LLT at universities and other higher education institutes, and even during staff training [4]. Currently, e-learning has brought about a new paradigm where learning is made possible in any field, for anyone, at any time, and from anywhere [5].

Although e-learning provides the potential for creating learner-centered learning environments and flexible learning approaches but Various research studies have highlighted the constraints of e-learning, such as the lack of face-to-face interaction, scheduling during non-optimal times, learners’ unfamiliarity with computer usage and peripheral services, limited access to information technology tools for learners, high costs associated with e-learning, weaknesses in learner assessment, and concerns regarding anxiety and insufficient effectiveness [6, 7].

Therefore, this question is always raised regarding the outcome of e-learning and the level of satisfaction among students, learners, and teachers with virtual education. Satisfaction, in this context, refers to the enjoyment and contentment a person experiences in their role and experiences as a user [8]. In other words, electronic satisfaction refers to the level of customer satisfaction with the design, content of information provided, and the delivery of appropriate services by electronic learning systems [9]. Thus, understanding the factors that affect the satisfaction of users, including learners and teachers, with these systems is of great importance, as it is a significant factor in measuring the quality of the teaching and learning process [10].

After all, many researchers have thus far devoted much attention to e-learning as a new paradigm. By way of example, Wing and Shi had proposed a novel model of user satisfaction based on the expectation-confirmation model (ECM), containing four key quality factors, viz., learning process, student-tutor interaction, peer interaction, and course design, in order to identify and develop students’ experiences with e-learning, which had then resulted in their satisfaction [11]. Using a quantitative research design and partial least squares structural equation modeling (PLS-SEM), Bervell et al., in their study on 322 participants also showed that the model for e-learning satisfaction was associated with teacher-student interaction (TSI), LLI, and LCT. Besides, LCT could have an effect on TSI and LLI development. Likewise, the structure and organization of training courses, student engagement, and LTI are among the factors affecting learner satisfaction with e-learning, thereby augmenting the retention and quality of e-learning and teaching [12]. Cole et al. had also presented the results of their three-year study on satisfaction with e-learning among students and graduates, revealing that they had shown moderate levels of satisfaction, although higher satisfaction had been seen regarding combined or relatively online courses. Of note, no interaction had been acknowledged as one of the major reasons for their partial dissatisfaction with e-learning [13].

In their study, Kurucay and Inan had also shown that LLI could have an effect on satisfaction with online education among learners. Thus, learners who had worked cooperatively had reached more success and satisfaction in this domain [14]. As stated by Ejubovic and Puska, goal-setting, metacognition, environmental structure, computer self-efficacy, and social dimension, as five central factors, could play a part in student satisfaction with online education, and subsequently their academic performance [15]. O’Connor et al. had correspondingly suggested that it was time, after years of academic research on e-learning, to create good environmental conditions by educational products and research support to encourage participants, users, and learners at universities to share experiences, collaborate, and hold group meetings [16].

The line of research during the last decade about e-learning systems implies that some focal determinants are likely to contribute to their success. In this respect, the internal and external motivation factors in learners, self-regulation, TSI, LLI, and course design have been so far mentioned, since they finalize the outputs of such systems [17]. As a web-based learning ecosystem, e-learning is typically practiced to disseminate information, launch communication, and transfer education and knowledge. Identifying the impact of e-learning on each society, like its benefits for the connection of e-learning systems, is accordingly correlated with the success and satisfaction of its users. What is more, the quality of system integration and information exchange exerts an influence on the success of such systems [18].

Although virtual systems can facilitate job monitoring and professional evaluation in the workplace, they also provide good opportunities for expanding scientific content and enhancing deep learning for learners in the work environment, as web-based training courses can be used as supplementary and complementary courses. This is due to the flexibility of electronic learning systems [19, 20].

On the other hand, the users of virtual learning systems include faculty members and teachers in universities and other higher education institutions, who play a key role in the teaching and learning process. Therefore, providing appropriate training courses can lead to further advancement of faculty members and teachers in familiarizing themselves with virtual learning systems and e-learning [21]. Jafari Far “et al.” have stated that one of the most significant barriers to the implementation of e-learning is insufficient familiarity of teachers with virtual learning systems [22].

Also, it is expected that students and the other learners will be able to keep up with organizational innovations and be prepared to achieve the predetermined organizational goals and attaining skill development and professional competencies through e-learning [23]. The training of learners’ on the job training, in terms of their social roles and characteristics, differs from universities students. They face challenges such as limited time for learning, work and family obligations, lack of access to training classes in the geographical area of their workplace, diversity among adult learners in terms of age and learning ability, motivational level and physical and mental preparedness, and differences in the assessment system for their acquired knowledge. All these factors influence the attitudes of this group towards organizational training and learning, making it significant [24].

Therefore, it appears that multiple factors influence users̓ satisfaction in e-learning. These factors have been categorized into six groups in most studies, including the learner, teacher, curriculum, technology, design, and environment. Even if online education has created a good opportunity for adult learners to learn continuously in their lifelong, there are still some challenges in educating these individuals [25]. In this context, Kara et al. had found that adult learners had been dealing with a number of challenges related to the internal and external factors in organizations, as well as the educational programs, age, gender, and knowledge and skills for using online educational tools and systems. Also, in an interview with the learners receiving online education, it was shown that learner satisfaction with distance learning and interaction had been at a moderate level. The study results had additionally established that distance learning and interactions between learner and the environment (here, computers) had led to cooperation and collaboration between learners and teachers [26]. In addition, Huang et al. had investigated the relationship between discourse, structure, and autonomy of learners in distance learning and interaction, and then reflected on environmental and demographic factors among those enrolled in web-based distance courses, and concluded that the structure and discourse of distance education were at a high level if the required support had been provided by policymakers and top-level managers at universities and educational institutions [27].

The future vision of education is generally shaped by e-learning. Developing the DeLone and McLean Information Systems Success Model (DMISM), Aldholay et al. had attempted to propose some independent variables, including technological, task-related, personal, and social characteristics along with realistic application, user satisfaction, and cognitive learning in order to design a guideline for policymakers and top-level managers of academic education [28].

Based on observations of virtual education at Beijing University, Bao and other researchers introduced six instructional strategies in 2020 to enhance learners’ focus and learning outcomes when transitioning to online learning. These strategies include:

1. Planning and preparedness for emergencies and unexpected crises.
2. Breaking down the instructional content into smaller units to assist learners’ focus.
3. Emphasizing the use of “voice” in online instruction.
4. Collaborating with teaching assistants for teaching and obtaining online support from them.
5. Enhancing learners’ self-directed learning abilities outside the classroom.
6. Effectively combining online learning with offline self-study [29–32].

Laying much focus on online education by universities has been one of the consequences of the coronavirus disease (COVID-19) pandemic in the world. As a result, training studios have been designed and constructed, and offline and online systems have been used to provide online education. Although such studios and systems at universities have been grappling with some challenges, users and learners have been in favor of online education and e-learning in this era [33].

In India, Urvashi Tandon had reported that the development of facilities in educational institutions could have a positive impact on learners’ attitudes and expectations with regard to e-learning during COVID-19. Although users’ social attitudes in the course of the pandemic could not be related to their views on online education, they had an effect on their tendency toward using online education facilities [34].

Ranjbar Kouchaksaraei “et al.” in a review study stated, the increased use of e-learning methods during the COVID-19 pandemic, and suggest that the educational approaches used during and after the control of COVID-19 should be given special attention so that all learners can benefit from them in the future in the event of similar crises [35].

The study conducted by Rajhans “et al.” (2020) in India during the COVID-19 pandemic showed that most educational activities are being carried out using video conferencing tools, dedicated virtual platforms, and social media. This research revealed that the rapid transition from traditional education to online mode has benefited students in the continuation of visual acuity assessment programs during the COVID-19 period and has effectively served them upon completion of their academic terms [36].

The study conducted by Chung “et al.” (2020) demonstrated that students, in general, have the necessary readiness for online learning and preferred virtual education through pre-recorded lectures on platforms such as Google Chrome and YouTube. However, the biggest challenge for postgraduate-level students was internet connectivity, while undergraduate students faced difficulties in understanding the content. This research suggested that universities should provide more training to enhance the virtual teaching skills of learners to ensure more effective learning outcomes [37].

Moreover, contextual factors (cultivation), inputs (virtual education infrastructure, appropriate content, learner and teacher capabilities), processes (teaching, assessment, monitoring and support, learner-teacher interaction), and outcomes (planning for improvement) are influential factors in the quality of virtual education. These factors should be taken into consideration during the COVID-19 pandemic period [38].

Li “et al.” concluded that the effectiveness of e-learning among students does not change over time. It was found that students’ preferences for face-to-face learning did not decrease after the subsiding of the COVID-19 pandemic. However, it can be believed that over time, students’ increased experiences with e-learning can lead to a more comprehensive perspective on blended learning, which includes both face-to-face and electronic components [39]. In their study conducted in China in 2021, Ming Tang “et al.” also stated that students’ readiness for online learning during the COVID-19 pandemic has not been well recognized, and various factors such as self-reliance, gender differences, and educational levels have played a key role in students’ motivation for e-learning during the corona virus period [40].

The main research question addressed was which aspects of e-learning and synchronous and non-synchronous systems of online education could meet student satisfaction at Tehran University of Medical Sciences, Tehran, Iran, during the COVID-19 pandemic, and how to strategically develop these systems in terms of infrastructure, technology, content, and LLI for the period of pandemics and crises and even as a combination in education for students and staff. Therefore, based on the aforementioned information, the present study was conducted with the aim of comparing the user satisfaction of virtual learning systems with the quality of synchronous and asynchronous systems during the COVID-19 period at Tehran University of Medical Sciences.

## Methods

The present research was descriptive-analytical study conducted cross-sectionally in Tehran University of Medical Sciences, from 2019 to 2022.

The research population in this study consisted of 8,928 undergraduate and doctoral students from various disciplines in selected faculties as well as 12,682 employees from the administrative units and headquarters of the university. The inclusion criteria for student participants were being enrolled in the undergraduate and doctoral programs in basic medical and allied medical sciences with a minimum of two years of study, familiarity with e-learning systems. For employees, the inclusion criteria were having at least a bachelor’s degree, types of employment status (temporary, or permanent), and a minimum of one year of work experience, as well as familiarity with the educational and e-learning systems. The exclusion criteria for both students and employees were a lack of willingness to participate in the study. The study was conducted from 2019 to 2022, during the COVID-19 pandemic, when all learners were required to use virtual systems for education and training.

The determination of the sample size was performed using the Krejcie and Morgan table, resulting in a sample size of 370 for students and 650 for employees. The reason for using the Krejcie and Morgan table was that the authors did not have access to the variance or the success rate of the statistical population. In this case, it was not feasible to implement the Cochran formula based on the limited or unlimited statistical population, as well as the measurement scales of the research variables [41]. The sampling method was proportionate stratified sampling, considering the research population and the availability due to the constraints imposed by the COVID-19 pandemic.

Data collection was carried out through a researcher-constructed web-based questionnaire, consisting of 24 questions on learner satisfaction with virtual learning systems, using a 5-point Likert scale ranging from “strongly agree” (score 5), “agree” (score 4), “agree to some extent” (score 3), “disagree” (score 2), to “strongly disagree” (score 1). The questionnaire was uploaded on the platform of “Porsline”.

The questionnaire was distributed among WhatsApp groups of learners, including students from selected faculties and employees from administrative units and the university’s headquarters, based on short-term on the job training courses. This was done with the coordination and permission of the educational deputies of selected faculties and the Staff Training Center of Tehran University of Medical Sciences. The objectives of the research were explained, and follow-ups were made via email, telephone, or in-person to complete the questionnaires. As a result, considering a 75% response rate coefficient, a total of 280 completed questionnaires were collected from students, and 500 completed questionnaires were collected from employees. The 75% response rate and the completion of the questionnaires by the statistical population of 8,928 students and 12,682 employees at Tehran University of Medical Sciences, Tehran, Iran, at the time of data collection through a short 24-item questionnaire, indicated the generalizability and high quality of the answers to the questionnaire items [42] in the present study.

To determine the level of satisfaction among students and employees with different types of educational systems and their components, the following criteria were used: satisfaction below 50% was considered low satisfaction, relative satisfaction was between 50% and 75%, and above 75% was considered high satisfaction.

The researcher-constructed questionnaire consisted of five dimensions regarding the use of e-learning and software deployment in terms of goals, instructional content, system users̓ specifications, conditions of the faculties and Staff Training Centers, and employees’ satisfaction. Each dimension was composed of several components, which were examined for their relationship with user satisfaction in virtual learning systems in the questionnaire. The dimensions and their respective components are as follows:

- Goals: Achieving educational objectives and expressing expectations.
- Educational or training Content: Appropriateness of the presented content volume, appropriateness of task volume, adequacy of allocated time for question and answer, provision of appropriate feedback on tasks and questions, and diversity of content.
- System User Specifications: Users’ electronic skills, users’ previous experience in virtual learning, users’ demographic characteristics, willingness to continue using e-learning after the COVID-19 pandemic, and overall satisfaction with virtual learning.
- Schools and Staff Training Center: Communication of policies and educational regulations, training on system usage, and support services.
- Software: User-friendliness, ease of system access, interaction among students or learners, interaction between instructors and students or learners, availability, easy access to instructional content, task uploading capability, question and answer functionality, and software flexibility.

The questionnaire were confirmed using Cronbach’s alpha coefficient, and a reliability coefficient of 75% was achieved. Along these lines, the internal consistency of the questionnaire items was confirmed. Otherwise stated, the value of 75%, between 70% and 80%, was acceptable for non-clinical studies.

To establish their validity, the face validity method was employed, and the questionnaire were given to 10 specialized faculty members in the fields of medical education, e-learning, and educational management, and received their approval. To confirm the content validity of the questionnaire in this study, the content validity ratio (CVR) was determined by nine experts and faculty members at the e-learning departments involved in medical education, the Center for Strategic Research in Medical Education, and the Department of Epidemiology and Biostatistics of Tehran University of Medical Sciences, Tehran, Iran. Accordingly, the CVR for the given questionnaire was 0.79. The content validity index (CVI) was also measured by a panel of experts in medical education and e-learning, whose value was equal to 0.81.

For data analysis, SPSS version 25 software was utilized, and descriptive statistics, including absolute and relative frequencies, were used to present the results. Additionally, independent t-tests, analysis of variance (ANOVA), and follow-up Post Hoc tests were employed at a significant level of 0.05 to present the analytical results. Using the independent t-test, the average of two independent communities is compared and a decision is made regarding their statistical difference. In our research, this test shows the significance of the relationship between satisfaction and other components. Analysis of variance (ANOVA) is used to analyze the average difference of different groups of research data. In the present study, the analysis of variance generalizes the t-test. Post Hoc test is also an integral part of ANOVA. In the current study, when the difference in means is significant, Post Hoc test is used to determine which of the groups these differences exist.

## Results

Based on the results shown in Table 1, it was determined that the highest satisfaction among students was related to “possessing electronic learning skills,“ while among employees, it was related to “high flexibility” and “willingness to use virtual learning after the COVID-19 pandemic.“ Additionally, the lowest level of satisfaction among students was related to “achieving the educational goals through e-learning,“ and among employees, it was related to “internet speed in virtual learning.“ According to the obtained results, it appears that the majority of students and employees have relative satisfaction with virtual learning and its components. Furthermore, using an independent t-test between the gender of students and employees and their satisfaction with virtual learning, a significant correlation was found (p < 0.05), although no correlation was observed between the satisfaction level of employees and students and their previous experiences in using virtual learning (p > 0.05). Based on the t-test, a significant correlation was found between the satisfaction level of learners (students + employees) and all components of virtual or electronic learning (p < 0.001), with effect size of 0.8 using variance analysis confirmed the high effect size between these two variables.

**Table 1:** Frequency distribution of learners’ satisfaction with virtual learning and its components during the COVID-19 pandemic

Component	Type of learners	Level of satisfaction with virtual learning		Statistical test result
		Satisfaction	Dissatisfaction	
		Number (percentage)	Number (percentage)	Pvalue
Expressing expectations and objectives at the beginning of a course	Employees	(62) 310	(38) 105	< 0/001
	Students	(49) 136	(51) 144	< 0/001
Achieving the educational goals	Employees	(49) 240	(51) 260	< 0/001
	Students	(41) 120	(59) 136	< 0/001
Availability of technical facilities and infrastructure	Employees	(34) 170	(66) 330	< 0/001
	Students	(51) 144	(49) 163	< 0/001
Enhancing the speed and effectiveness of virtual learning in education	Employees	(57) 280	(43) 220	< 0/001
	Students	(51) 144	(49) 136	< 0/001
High flexibility	Employees	(79) 400	(21) 100	< 0/001
	Students	(57) 160	(43) 120	< 0/001
Internet speed	Employees	(21) 100	(79) 400	< 0/001
	Students	(46) 130	(54) 150	< 0/001
Possessing electronic skills by learners	Employees	(64) 320	(36) 180	< 0/001
	Students	(76) 215	(24) 130	0/026
Overall satisfaction with virtual learning	Employees	(70) 350	(30) 150	< 0/001
	Students	(51) 144	(49) 136	< 0/001
Willingness to use virtual learning after the COVID-19 period	Employees	(83) 420	(7) 80	0/004
	Students	(54) 150	(46) 130	< 0/001

In examining the correlation between age and employees satisfaction with virtual learning, a one-way analysis of variance (ANOVA) test was conducted, resulting in a significant correlation (p = 0.03), with effect size of 0.5 using variance analysis confirmed the moderate effect size. This finding provided evidence of a meaningful relationship within this group of learners regarding their satisfaction with the training. To further investigate the relationship between employee’s satisfaction and age groups, POST HOC as follow up exam test was utilized. As a result, a significant correlation was observed between satisfaction with virtual learning and age groups of 21–30, 31–40, and 40 and above. It was concluded that employee satisfaction increases as age increases.

Furthermore, by employing an analysis of variance, it was determined that there is a significant correlation between students satisfaction with virtual learning and their age (p = 0.002). As age increases, the level of student’s satisfaction with electronic learning also increases.

To compare the overall satisfaction of employees and students at Tehran University of Medical Sciences regarding electronic learning, a t-test was performed, revealing a significant difference in the level of satisfaction between these two user groups (p = 0.031), with effect size of 0.6 using variance analysis confirmed moderately effect size between these two variables. It was evident that employee’s satisfaction with virtual learning was moderately higher than that of students.

According to Table 2, the highest level of satisfaction among the university students was related to the “effective feedback on assignments,“ while the lowest level of satisfaction was associated with the “user-friendliness of the platform’s appearance.“ However, university employees expressed the highest level of satisfaction with the “interaction among learners” component and the lowest level with the components of “user-friendliness of the platform’s appearance” and “easy access to the system.“

**Table 2:** Frequency distribution of users satisfaction with the navid system based on its components during the COVID-19 pandemic

Component	Type of learners	Satisfaction level with the Navid system and its components		Statistical test result
		Satisfaction with the component	Dissatisfaction with the component	
		Number (percentage)	Number (percentage)	Pvalue
User-friendliness of the platform’s visual design	Employees	(15) 70	(85) 430	0/02
	Students	(16) 30	(84) 250	0/04
Easy access to the system	Employees	(15) 70	(85) 430	0/005
	Students	(19) 50	(81) 230	0/006
Appropriate volume of provided content	Employees	(30) 150	(70) 350	< 0/001
	Students	(38) 106	(62) 174	< 0/001
Instant access to content	Employees	(26) 130	(74) 370	< 0/001
	Students	(27) 80	(73) 200	0/004
Easy access to support services	Employees	(40) 200	(60) 300	< 0/001
	Students	(68) 188	(32) 92	0/135
Appropriate workload	Employees	(40) 200	(60) 300	0/001
	Students	(57) 160	(43) 120	< 0/001
Appropriate dedicated time for completing assignments	Employees	(34) 170	(66) 330	0/001
	Students	(43) 120	(57) 160	< 0/001
Effective feedback on assignments	Employees	(57) 280	(43) 220	0/22
	Students	(81) 225	(19) 55	< 0/001
Variety of content presented	Employees	(36) 180	(64) 320	< 0/001
	Students	(49) 135	(51) 145	< 0/001
Interaction among learners	Employees	(74) 370	(26) 130	0/007
	Students	(62) 175	(38) 105	< 0/001
Interaction between learners and teachers	Employees	(66) 330	(34) 170	0/003
	Students	(54) 150	(46) 130	< 0/001
Need for more time	Employees	(21) 100	(79) 400	0/07
	Students	(46) 130	(54) 150	0/001

Using the t-test at a significance level of P < 0.05, a significant difference in satisfaction was found between learners (students and staff) regarding the components of the Navid system, except for “need for more time,“ “easy access to support services,“ and “effective feedback on assignments.“ Additionally, no significant difference in satisfaction levels between employees and students was observed regarding the offline Navid system (p = 0.171). Overall, it seems that the satisfaction level of both students and employees with the offline Navid system is below 50%, indicating a low level of satisfaction among the learners with this system.

To establish a significant correlation between user’s satisfaction with the Navid system and factors such as gender, age, and previous experiences with virtual learning, a t-test was conducted. The results did not show a significant correlation between user’s satisfaction and learners’ satisfaction with the Navid system and their gender or previous experiences (p > 0.05). However, a significant correlation was observed between the age of students and their satisfaction level with the Navid system using a one-way analysis of variance (ANOVA) test (p < 0.01), with effect size of 0.6 using variance analysis. To determine which specific age groups exhibited this difference, POST HOC follow up test was employed. The analysis revealed that satisfaction with the Navid system increases as students’ age increases.

Based on Table 3 and considering that only the participating students used the Big Blue Button system in this study, it can be concluded that the highest level of student satisfaction was related to the “appropriate volume of educational content”, while the lowest level of satisfaction was related to “access to support services” of the system.

**Table 3:** Distribution of student satisfaction with the BigBlueButton learning management system and its components during the COVID-19 pandemic

Component	level of satisfaction with BigBlueButton and its components		Statistical test result
	Satisfaction with the component	Dissatisfaction with the component	
	Number (percentage)	Number (percentage)	Pvalue
user-friendliness of the platform’s interface	(53) 150	(47) 130	< 0/001
easy access to the system	(60) 170	(40) 110	0/006
appropriate volume of educational content	(73) 205	(27) 75	< 0/001
instant access to content	(53) 150	(47) 30	0/28
access to support services	(20) 55	(80) 225	0/004
appropriate time for asking and answering questions	(40) 110	(60) 170	0/001
feedback on questions and answers within the system	(47) 130	(53) 150	0/01
interaction between the instructor and the student	(40) 110	(60) 170	0/004
time flexibility compared to in-person education	(47) 130	(53) 150	0/001
internet speed	(67) 190	(33) 90	0/36
increased learning due to online nature	(53) 150	(47) 130	0/001

Furthermore, a significant correlation was observed between student satisfaction and all components of the Big Blue Button system except for “instant access to content” and “internet speed” (p < 0.05), with effect size of 0.6. There was no significant correlation found between gender, age, previous experiences with the Big Blue Button system, and student satisfaction, as determined by the t-test (p > 0.05). Additionally, the use of an ANOVA test revealed that there is no significant difference in satisfaction levels between different age groups and the Big Blue Button System (p > 0.05). Moreover, the overall satisfaction rate of students with the mentioned system was less than 50%. In conclusion, it appears that students have low satisfaction with the online Big Blue Button System and its components.

Based on the results obtained from Tables 4, the highest level of employees satisfaction was related to the “user-friendliness of the platform’s appearance,“ while the lowest level of satisfaction was associated with the “time-consuming nature of the system.“ Among the participating students in this study, the highest level of satisfaction was derived from the “internet speed,“ whereas the lowest level of satisfaction was attributed to “Easy access to content at all times.

**Table 4:** Distribution of satisfaction levels among students and employees with adobe connect system and its components during the COVID-19 pandemic

Component	Type of learners	Satisfaction level with the Adobe Connect System and its components		Statistical test result
		Satisfaction with the component	Dissatisfaction with the component	
		Number (percentage)	Number (percentage)	Pvalue
User-friendliness of the platform’s visual design	Employees	(78) 390	(22) 110	0/30
	Students	(39) 110	(61) 270	0/02
Easy access to the system	Employees	(56) 280	(44) 220	0/09
	Students	(46) 130	(54) 250	0/001
Instant access to content at all times	Employees	(33) 165	(67) 335	0/104
	Students	(21) 80	(79) 200	0/003
Easy access to support services	Employees	(56) 280	(44) 220	0/02
	Students	(56) 160	(44) 220	0/01
Appropriate timing for presenting questions and answers	Employees	(67) 340	(33) 160	0/119
	Students	(39) 110	(61) 270	< 0/001
Effective feedback within the system for questions and answers	Employees	(55) 265	(45) 235	0/02
	Students	(50) 140	(50) 140	< 0/001
Interaction between teachers and learners	Employees	(44) 220	(56) 280	0/04
	Students	(50) 140	(50) 140	< 0/001
Time consumming nature of the system	Employees	--	(100) 500	--
	Students	(46) 130	(54) 150	0/002
Internet speed	Employees	(56) 280	(44) 220	0/69
	Students	(61) 170	(39) 110	0/05
Increase in learning due to online nature	Employees	(22) 110	(78) 390	0/16
	Students	(25) 70	(75) 210	< 0/001

A meaningful correlation was observed between the satisfaction levels in the components of “easy access to support services” (P = 0/02), “interaction between teachers and learners” (P = 0/04), and “effective feedback within the system for questions and answers (P = 0/02) in both groups, with effect size of 0.6 using variance analysis confirmed moderately effect size between variables for all. Although a meaningful correlation was found between the satisfaction levels among students and all the components of the Adobe Connect system (P < 0/05), but this was not the case for employees.

Furthermore, no meaningful correlation was found between gender, age, previous learning experiences, and the satisfaction levels of learners with the Adobe Connect system using a t-test (P > 0/05).

To identify meaningful differences among users satisfaction levels with the Adobe Connect online system among different age groups, an ANOVA test was employed, but no significant differences were observed in this regard (P > 0/05).

Based on the results obtained from Table 5, the highest level of employees satisfaction was related to the components of “user-friendliness of the platform’s visual design,“ “appropriate timing for submitting questions and answers,“ “interaction among learners,“ and “time consuming system.“ On the other hand, the lowest level of satisfaction was associated with the components of “Instant access to content at any time” and “easy access to support services.“ Among the students, the highest level of satisfaction with the components was related to “easy access to the system,“ while the lowest level was associated with “easy access to support services.“

**Table 5:** Frequency distribution of students’ and employees satisfaction with the skyroom system and its components during the COVID-19 pandemic

Component	Type of learners	Satisfaction level with Skyroom		Statistical test result
		Satisfaction with the component	Dissatisfaction with the component	
		Number (percentage)	Number (percentage)	Pvalue
User-friendliness of the platform’s visual design	Employees	(64) 350	(36) 200	< 0/001
	Students	(82) 230	(18) 50	0/007
Easy access to the system	Employees	(55) 300	(45) 250	0/06
	Students	(93) 260	(7) 20	0/02
Appropriate volume of educational content	Employees	(58) 320	(42) 230	< 0/001
	Students	(86) 240	(14) 40	0/008
Instant access to content at any time	Employees	(48) 240	(52) 290	< 0/001
	Students	(57) 16	(43) 120	< 0/001
Easy access to support services	Employees	(48) 160	(52) 290	< 0/001
	Students	(46) 260	(54) 150	0/04
Appropriate timing for submitting questions and receiving answers	Employees	(64) 350	(36) 200	< 0/001
	Students	(71) 200	(29) 80	0/001
Appropriate feedback within the system for questions and answers	Employees	(58) 320	(42) 230	< 0/001
	Students	(75) 210	(25) 70	< 0/001
Interaction among learners	Employees	(64) 350	(36) 200	< 0/001
	Students	(68) 190	(32) 90	< 0/001
Interaction between teachers and learners	Employees	(58) 320	(42) 230	< 0/001
	Students	(75) 210	(25) 70	0/003
Time consuming system	Employees	(64) 350	(36) 200	0/74
	Students	(75) 210	(25) 70	0/45
Internet speed	Employees	(58) 320	(42) 230	0/007
	Students	(68) 190	(32) 90	0/31
Increased learning due to being online	Employees	(61) 340	(39) 210	0/005
	Students	(71) 200	(29) 80	0/003

By conducting a t-test on a group of students and considering P < 0.05, a significant correlation was found between students satisfaction with all components of the SkyRoom System except for “time consuming system” and “internet speed”. Similarly, among the employees, a significant correlation was observed between employees satisfaction and other components of the SkyRoom System, except for " time consuming system " and “easy access to the system,“ based on the t-test with P < 0.05, with effect size of 0.8.

To examine the relationship between learners’ satisfaction with the SkyRoom System and gender, experience, and previous experiences in virtual training, a t-test was employed. It was determined that among the students, there was no significant difference in satisfaction with the SkyRoom System based on demographic factors, given P > 0.05. However, among the employees, a significant difference in satisfaction with the SkyRoom System was observed based on their previous experiences with virtual training (P = 0.02). This indicates that users with prior experiences with virtual training through this system are more satisfied compared to those who are using the system for the first time.

By conducting a t-test with P = 0.02, a significant difference in satisfaction between the two user groups was observed, with students exhibiting higher satisfaction with the SkyRoom System compared to employees, with effect size of 0.6. However, both students and employees expressed moderately high satisfaction with the online SkyRoom system in e-learning.

According to Tables 6, the highest level of employees satisfaction was related to “easy access to the system,“ while the lowest satisfaction level was associated with “Time consuming system.“ Similarly, students expressed higher satisfaction with the component of “easy access to the system” and lower satisfaction with “Time consuming system.“

**Table 6:** Frequency distribution of satisfaction levels of students and employees with skype software and its components during the COVID-19 pandemic

Component	Educational institution	Satisfaction level with the Skype software		Statistical test result
		Satisfaction with the component	Dissatisfaction with the component	
		Number (percentage)	Number (percentage)	Pvalue
User-friendliness of the platform’s appearance	Employees	(74) 410	(26) 70	0/03
	Students	(83) 240	(17) 40	0/006
Easy access to the software	Employees	(97) 520	(3) 60	0/85
	Students	(93) 260	(7) 20	0/20
Appropriate volume of educational content	Employees	(82) 450	(18) 100	< 0/001
	Students	(62) 180	(38) 100	< 0/001
Instant access to the content	Employees	(84) 471	(16) 39	0/002
	Students	(83) 240	(17) 40	0/01
Appropriate timing for presenting questions and answers	Employees	(83) 460	(17) 90	< 0/001
	Students	(79) 220	(21) 60	< 0/001
Appropriate feedback in the system to questions and answers	Employees	(66) 370	(34) 180	< 0/001
	Students	(66) 190	(34) 90	< 0/001
Interaction among learners	Employees	(58) 320	(42) 230	< 0/001
	Students	(76) 210	(24) 70	< 0/001
Interaction between the instructor and learners	Employees	(55) 300	(45) 250	< 0/001
	Students	(76) 210	(24) 70	< 0/001
Time consuming system	Employees	(34) 200	(66) 350	0/1
	Students	(41) 120	(59) 160	0/07

Through conducting t-tests in each group of learners and considering the significance level of P < 0/05, with effect size of 0.8, a significant relationship was observed between the satisfaction levels of students and employees with all the software components of Skype, except for the components of " Time consuming system " and “easy access to the software.“

To determine the relationship between users’ satisfaction with the Skype software and their gender, age, and previous experiences with virtual learning, t-tests were used, which indicated that no significant relationship was found with a significance level of P > 0/05.

Furthermore, to find a significant relationship between the satisfaction levels of employees and students in different age groups, ANOVA tests were employed, revealing that no significant relationship was observed in any age group (with a significance level of P > 0/05).

By conducting a t-test with a significance level of P = 0.142, no significant difference in satisfaction levels was observed between these two user groups of Skype.

As observed in the results of Table 7, employees and students of Tehran University of Medical Sciences expressed a relatively satisfaction with virtual learning during the COVID-19 pandemic. Although there was a significant difference in satisfaction between these two groups of learners regarding virtual education, with employees who were simultaneously undergoing training courses while working showing higher satisfaction compared to students (p = 0.031), with effect size of 0.6. Furthermore, the significant difference in satisfaction between these two groups of learners regarding virtual education was confirmed through the POST-HOC test (p = 0.02).

**Table 7:** Distribution of satisfaction levels among students and employees based on virtual education, platforms, and software during the COVID-19 pandemic

Type of education, platforms, and software	Type of learners	Level of satisfaction				Stages of satisfaction	Statistical test result
		Satisfaction		Dissatisfaction			
		Number	Percent	Number	Percent		p-value
Virtual education (Offfline + Online)	Employees	288	52	262	48	Relative dissatisfaction towards deterioration	0/031
	Students	150	53	130	47	Relative dissatisfaction towards deterioration	
Asynchronous education (Navid)	Employees	188	67	362	33	Relative satisfaction	0/171
	Students	129	46	151	54	Low satisfaction	
Big blue button	Employees	-	-	-	-	-	-
	Students	141	51	139	49	Relative dissatisfaction towards deterioration	
Adobe connect	Employees	259	47	291	53	Low satisfaction	0/197
	Students	124	44	156	56	Low satisfaction	
Skyroom	Employees	320	58	230	42	Relative satisfaction	0/02
	Students	202	72	78	28	Relative satisfaction towards improvement	
Skype	Employees	389	70	161	30	Relative satisfaction towards improvement	0/142
	Students	207	73	73	27	Relative satisfaction towards improvement	
Online education	Employees	327	60	223	40	Relative satisfaction	0/954
	Students	168	60	112	40	Relative satisfaction	

In addition, selected faculty students expressed a low satisfaction with asynchronous learning (Navid system), while employees showed relative satisfaction with the Navid system. However, no significant difference in satisfaction between these two groups of learners during the COVID-19 pandemic was observed regarding the Navid system as an asynchronous e-learning platform (p = 0.171).

Both groups of learners (students + employees) expressed relative satisfaction with various online learning systems and software during the COVID-19 pandemic at the university, although no significant difference in satisfaction between these two groups was found (p = 0.954).

Among the various online learning systems and software, the highest level of satisfaction reported by both employees and students was related to the Skype System, although no significant difference in satisfaction between these two groups of learners regarding this system was identified (p = 0.142).

The lowest level of satisfaction among learners was associated with the Adobe Connect System for online education. However, no significant difference in satisfaction between students and employees regarding this system was reported (p = 0.197).

Among the components of virtual education, asynchronous training (Navid system) and online training the highest level of user satisfaction was related to system and software flexibility, effective feedback in questions and answers, interaction between teachers and faculty members and learners, provision of support services, and user-friendliness of the platform. The lowest level of satisfaction was associated with internet speed and system downtime.

Among demographic characteristics, the age of learners was a factor that showed a significant relationship with the satisfaction of learners with online education, asynchronous learning (Navid system), and overall electronic education or e-learning. As age increased, learner satisfaction with learning and electronic education also increased (p = 0.002, p = 0.01). However, no significant relationship was observed between learner satisfaction and gender or previous experience with electronic learning systems (p > 0.05).

## Discussion

The results obtained from the present study indicated that students from selected faculties and employees of Tehran University of Medical Sciences expressed relative satisfaction with virtual education, especially online learning, during the COVID-19 pandemic. However, employees who were undergoing on the job training during this period expressed higher satisfaction compared to students with electronic learning or education. In 2021, Zeng and Wang reported that University of Texas students were satisfied with asynchronous and online learning during the COVID-19 pandemic [43]. Additionally, Jamieson in 2020 [44] and Dickenson and Gronseth in 2020 reported students satisfaction with online and asynchronous electronic education [45]. Malkavi et al. (2020) reported student satisfaction in Jordan [46], while Hasan et al. (2020) reported low student satisfaction with e-learning in Saudi Arabia [47]. However, Naik et al. in 2021 conducted research on 874 students from different universities in India during the COVID-19 pandemic and reported low students satisfaction with electronic learning and education [48]. Yazdani Nezhad et al. (2020) were able to report low students satisfaction with medical universities during the COVID-19 pandemic in Iran [49]. The results of the present study somewhat coincide and are similar to the results of other studies conducted on this subject. Despite the necessity and need for electronic learning during the COVID-19 pandemic, students satisfaction with electronic education differs among different countries for various reasons.

Regarding the e-learning of employees or adults during the implementation of on the job training, the findings of Shams and colleagues among the employees and managers of the National Gas Company headquarters indicated that these individuals had a low satisfaction with e-learning [50]. Mohammadi et al. also reported at Hamedan University of Medical Sciences that the employees of this university expressed low satisfaction with e-learning on the job training courses for various reasons [51], although Biglar et al. reported high satisfaction of the employees of Tehran University of Medical Sciences who were undergoing orientation or socialization and on the job training courses during the COVID-19 pandemic [52]. Niknami et al. at the Organization for Research and Compilation of Humanities Books (SAMT) stated that traditional methods for enhancing empowerment and on the job training of employees were not fully effective during the COVID-19 pandemic and that there was a need for a practical and systematic design in virtual training levels and electronic systems [53]. Fengchun et al. also pointed out that e-learning during the COVID-19 pandemic was considered a guarantee for the effectiveness of empowerment and on the job training of employees [54]. It seems that the results obtained from this study are consistent with the findings of previous research regarding the effectiveness and satisfaction of employees with e-learning in on the job training.

Lischer et al. also found that both students and employees during the COVID-19 pandemic were satisfied with e-learning, although they stated that hybrid education during the COVID-19 pandemic can be more effective than e-learning alone [55]. These findings align with the results of the current study regarding the relative satisfaction of students and employees at Tehran University of Medical Sciences with e-learning. However, based on the findings of the current study, it was evident that employees had a higher level of satisfaction with e-learning during the COVID-19 pandemic compared to students. In this regard, the results of a comparative study by Taghizadeh et al. demonstrated that online systems provided more opportunities for employees compared to students during the COVID-19 pandemic, facilitating simultaneous completion of job-related tasks and learning [56]. Shokrzadeh and Asadzadeh also declared in their research during the COVID-19 pandemic that e-learning had become the primary and fundamental method for training employees in organizations, with employees playing a central role in this type of education and being able to effectively grasp complex subjects with more time dedicated to learning [57].

Although the current study did not observe a significant differences in satisfaction between employees and students regarding online and offline systems in e-learning during the COVID-19 pandemic, employees expressed higher satisfaction with the offline system compared to selected university students. Additionally, both groups reported relative satisfaction with online learning systems during this period. Zho and Zhang, in their research among college students in the United States during the COVID-19 pandemic, found that the use of online systems in e-learning was successful [58]. Sims and Baker announced in 2020 during the COVID-19 pandemic that student engagement and performance were reduced with online education compared to face to face education [59]. Young and Bruce conducted their research among undergraduate students in a university located in the Rocky Mountains region of the United States in 2020 during the COVID-19 pandemic and determined that participants in their study preferred face-to-face education over online education and had minimal satisfaction with online learning, thus indicating a preference for hybrid education during crises and potential future pandemics [60]. Yazdani Nezhad et al. also concluded that learners were not highly satisfied with online learning systems in the COVID-19 crisis [49]. Zheng and Wang demonstrated at the University of Texas in the United States that students during the COVID-19 pandemic used online and offline e-learning methods based on their applicability and effectiveness, expressing satisfaction with both approaches [43]. Jamison, Dickinson, and Gronseth also showed that learners had higher satisfaction with online systems [44, 45]. The results of the current study regarding satisfaction with online learning systems seem to align with the findings of other research. However, it should be noted that some studies have recommended the use of hybrid education among students.

The obtained results of the present study identified factors that could have influenced the satisfaction of learners in online and offline electronic education systems during the COVID-19 period at Tehran University of Medical Sciences. These factors included slow internet speed, incomplete achievement of educational objectives especially among students, ineffective support services, and time-consuming nature of some learning systems, which contributed to low satisfaction levels. On the other hand, factors such as learners’ electronic skills, flexibility of certain systems in the learning process, appropriate feedback, and effective interaction between learners and instructors were considered as factors leading to high satisfaction levels. In this regard, the research results of Mosavi Sahebolzamani et al. among the students of Zanjan University of Medical Sciences during the COVID-19 period revealed the presence of inappropriate technical and support structures [61], accurate and comprehensive educational content [37], negligence toward infrastructure, excessive workload, particularly regarding employee training, and poor planning in providing educational services in the electronic education system, which resulted in reduced access to faculty members and educators [56]. Additionally, technical, practical, infrastructural, and content-related weaknesses [62], insufficient interaction between students and educators [58], lower interaction compared to face to face education [59], lack of proficiency in using educational and e-learning systems [51], inadequate support services, imbalanced task volume and time [47], lack of facilities, infrastructure, technical tools, and limited internet access [48], hardware and software problems in internet connectivity [63], reduced quality and inadequate provision of support services, as well as insufficient response and support from educators [49],, untimely feedback and prolonged response time from educators [64], and insufficient response from educators [43] were among the factors that negatively influenced learner satisfaction. These findings are somewhat consistent with the conditions, technological advancements, and IT development of different countries. Although high interaction between faculty members and students and other learners, diversity in styles and content in virtual learning systems [65], flexible design of system platforms and opportunities for e-learning utilization [56], increased familiarity and professionalism of users with e-learning [66–69], workload and task volume balancing during e-learning [47], flexibility in e-learning time [1], high flexibility in the use of electronic systems [70], high proficiency among users, especially students [51], flexibility in content viewing and participation in asynchronous discussions [44, 45], user-friendly electronic systems [64], users’ high experiences, especially students, in using electronic systems [70, 71], were identified as factors that positively influenced the increase in learner satisfaction, which aligns with the findings of the present study.

One of the significant findings from the present study is the meaningful relationship between the age of learners, including students and employees, and their satisfaction with online and offline systems used during the COVID-19 pandemic at Tehran University of Medical Sciences. It has been identified that with increasing age, the satisfaction level of learners, especially among employees, is higher with online and offline systems. Although the results of the study by Mosavi Sahebolzamani et al. showed no meaningful relationship between students’ satisfaction with e-learning and their age [61]. However, Hilton et al. stated that younger students in their early academic years with less experiences working with online learning systems exhibit less satisfaction with online education and preferred traditional face-to-face and attendance learning [71].

All the conducted studies are to some extent consistent with the results of the present study, indicating that factors such as newly enrolled students in faculties with less experiences working with online systems have less satisfaction with virtual education. On the other hand, students with higher academic experiences or those who are simultaneously working while studying, as well as among the employees receiving training during their services, primarily in the age range of 30–40 years, exhibit higher satisfaction with e-learning and online/offline systems.

In the present study, no meaningful relationship was observed between learners’ satisfaction, including students and employees, and their gender or previous experiences with virtual learning systems. Hassan “et al.” also did not report any significant differences in the satisfaction of Saudi Arabian university students with virtual education based on their gender during the COVID-19 pandemic [47]. Bayrak et al. and Farsi et al. also reported consistent results with previous studies, stating that gender did not have an impact on learners’ satisfaction with electronic systems [70]. However, in the research conducted by Gonzalez “et al.”, contrary to the results obtained in the present study and the other similar studies, they reported a meaningful differences between gender and learners’ satisfaction with e-learning, favoring women [72].

Another important point is the use of facilitating services and technologies through which the use of virtual education systems can be promoted and thereby help increase the satisfaction of learners. Facilitating services are include as hosting web pages, delivering storage and processing capacities, hosting organization’s servers and etc. In this field, there are various companies that provide information technology services to users and other companies [73]. The use of new intelligent systems and algorithms in order to improve processes and functions related to processing information and communication and providing better services can help to improve virtual education systems and reduce the costs of using these systems and related programs [73, 74]. Cloud computing is one of these methods that has many advantages, including better processing and storage of information and improving communication [73–76]. The results of the present study also emphasize the importance of improving processes and functions related to information and communication processing, which can help increase the learner’s satisfaction of virtual learning systems.

This study has several unique characteristics. Firstly, it was conducted during the COVID-19 pandemic at Tehran University of Medical Sciences. It aimed to measure the satisfaction level of learners, including students and employees, with all the authorized online and offline educational systems and their components that were practically used by the learners during that period. Secondly, the study was simultaneously conducted on undergraduate students from selected faculties and university employees who were undergoing on the job training, orientation or socialization training courses, and professional and occupational empowerment courses. Therefore, it was a comparative study, considering factors such as age, gender, previous experiences with the mentioned systems, between students learning and adult learning. Thus, all positive and negative influential factors on learners’ satisfaction were introduced through electronic learning via online and offline systems. Consequently, necessary and appropriate solutions were proposed to enhance and improve the infrastructure, technical and support factors, software, and hardware in potential critical situations and future pandemics.

However, this study has certain limitations. Firstly, it assessed user’s satisfaction through self-evaluation using a questionnaire. Secondly, this study was conducted specifically at Tehran University of Medical Sciences, and the obtained results may not be generalizable to other medical universities in the country due to differences in technical, support, service, and infrastructural dimensions. Thirdly, there is a possibility that the outcomes of this study may not be similar to the findings in future crises, pandemics, and possible epidemics that necessitate the use of electronic learning.

## Conclusion

Certainly, there is no doubt that the COVID-19 pandemic has presented a beneficial chance for students and faculty members and the other educators in universities and other educational institutions to improve their abilities and adopt a positive mindset towards electronic learning, given the exceptional circumstances experienced in the country. However, it should be acknowledged that in certain instances, students have suffered significant adverse effects such as physical exhaustion, mental strain, and academic fatigue. A significant point during this period of study was the absence of any discrimination between educating students and employees. Both groups received equal and similar services in terms of infrastructure, technical support, and content. While learners in the present study expressed relative satisfaction with e-learning systems, it is essential to strengthen the infrastructure and provide continuous technical support, continuous updates, and improvement of educational and learning platforms by allocating more financial resources and developing policies and programs for improving digital services by both the government and senior administrators and policymakers of the education sector, and that seems to be necessary. By empowering and strengthening e-learning at Tehran University of Medical Sciences, it is hoped that these systems will continue to be utilized even in critical situations, in conjunction with hybrid in-person learning, thereby providing greater benefits. The use of facilitating services and technologies and practical and intelligent systems and programs to improve processes and functions related to information processing and storage and improving virtual communication should be considered in planning and formulating goals, strategies, and policies and the allocation of e-learning resources, in the future.

## Data Availability

The datasets used and/or analyzed during the current study are available from the corresponding author on reasonable request.
